# Reverse Objective Structured Clinical Examination (ROSCE)

**Published:** 2018-11-12

**Authors:** Tripti Srivastava, Lalitbhushan Waghmare

**Affiliations:** 1 Professor, Department of Physiology, Datta Meghe Institute of Medical Sciences (Deemed University), India; 2 Dean, Interdisciplinary Health Sciences, Datta Meghe Institute of Medical Sciences (Deemed University), India

## Introduction

Objective Structured Clinical Examination (OSCE) is a comprehensive, systematic, and objective method of assessment that involves an individual student rotating through a number of practical and theoretical “stations” where they are assessed using a set criterion.^1-3^ Better reliability, objectivity and assessment of Psychomotor, Communication and Affective domains are some of its advantages as an assessment tool.^2^

The authors propose a novel form – “Reverse OSCE (ROSCE)” which is build upon the principles of conventional OSCE. It is a step forward wherein the roles of tutor and student are reversed. ROSCE as an assessment tool enable assessment of observation skills of a medical student. “Observation” is the action or process of observing something or someone in order to gain information. Observation skills are an integral aspect of health professionals that hone their ability to deal with patients and respond in an appropriate manner, enabling them to make better clinical decisions.

### Specifics

Essentially, the principle of multiple stations, assessment of multiple domains, standardised patient and constant duration at each station are alike as in a conventional OSCE. The hallmark is role of tutor/teacher who demonstrates/performs a given task (on a particular station) and the student observes the task being performed. Thus, the roles of student and tutor are reversed. The student keenly observes and records his/her observations in a simple format (observation record sheet – 1, see Appendix A). The student has to record any mistakes, deviations and/or missing steps within the performed activity compared to standard practice. Technicians, residents and faculty can be the demonstrators. The ability to observe and analyse procedural, communication, physical examination, counselling skills etc. can be assessed by this method.

Another significant attribute is the inclusion of the last station as a “Reflect station.” Here the students reflect upon their observations from multiple stations, defend their observations and mention the correct approach compared to the mistakes recorded. The same “Observation record sheet” is to be used for reflection.

Group feedback is given after ROSCE wherein student’s observations and reflections with respect to each station are discussed. The emphasis is on the significance of keen observation in medical profession, the key aspects that should not be missed/ignored while performing given skills and the effect on learning by reflecting on action.

The number of skills, stations, checklist, and the step which is to be missed and/or incorrectly performed are pre-decided. Scores for identification and detailing of each missed and/or incorrect step is pre-decided. One or few stations in Reverse OSCE can have pre-recorded video clipping of a particular procedure or clinical scenarios. The student can watch the clip and record their findings.

### Attributes of Reverse OSCE

1. It can assess 8-10 students per ROSCE station at a given time.
2. It aids in sharping observation skills of learner and enable its assessment.
3. Enable assessment of reflective skills.
4. Technicians, residents and faculty members can take the role of demonstrators.

### Process of Reverse OSCE

**Figure 1:**
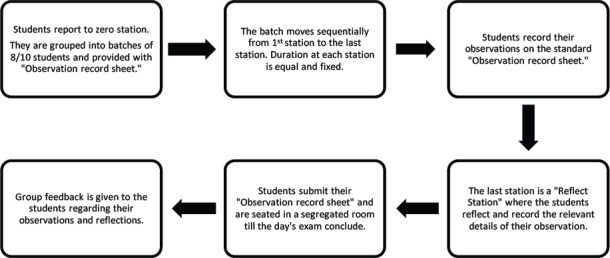
Process of implementation of ROSCE (Copyright Reg. no L-702245/2017)

The proposed process conduction of ROSCE is depicted in Figure 1.

### Conclusion

We have implemented the ROSCE as a part of the formative examination of 1^st^year professional students in the faculty of medicine. For outcome analysis of this new tool, marks obtained by students will serve as a measure of student’s observation skill and identify gaps in knowledge. The feedback from learners is encouraging and teachers have endorsed its attributes, however; the students need to be trained about how to write “reflections” beforehand. It is important to realise that ROSCE should not be considered an alternative to conventional OSCE. Rather, the ROSCE is different where learners have attained a certain level of competency and are aware of the standard methods of performing given set of skills and therefore can be assessed on other professional skills like observation and reflections.

## Appendix A Observation Record Sheet

Observation Record Sheet Roll Number: Subject:

**Table:**

Station no.	Skill observed	Deviations/mistakes/missed steps/any other observation	Reflections (discuss your observations and corrective actions)	Score (to be filled by examiner)
1				
2				
3				
4				
5				

Global scoring (to be filled by examiner):
